# Catheter Ablation vs. Standard Implantable Cardioverter Defibrillator Therapy in Symptomatic Brugada Syndrome: A Systematic Review and Meta-Analysis of Controlled Studies

**DOI:** 10.3390/medsci13030115

**Published:** 2025-08-06

**Authors:** Paschalis Karakasis, Panagiotis Theofilis, Konstantinos Pamporis, Antonios P. Antoniadis, Nikolaos Fragakis

**Affiliations:** 1 Second Department of Cardiology, Hippokration General Hospital, Aristotle University of Thessaloniki, 54642 Thessaloniki, Greece; aantoniadis@gmail.com (A.P.A.); fragakis.nikos@googlemail.com (N.F.); 2 First Department of Cardiology, School of Medicine, Hippokration General Hospital, National and Kapodistrian University of Athens, 15772 Athens, Greece; panos.theofilis@hotmail.com (P.T.); konstantinospab@gmail.com (K.P.)

**Keywords:** BRUGADA syndrome, catheter ablation, ventricular fibrillation, implantable cardioverter-defibrillator, sudden cardiac death

## Abstract

**Background**: Catheter ablation of the arrhythmogenic substrate has emerged as a promising therapeutic strategy for symptomatic Brugada syndrome (BrS). However, high-quality comparative evidence against conventional implantable cardioverter-defibrillator (ICD)-based management remains limited. **Objectives**: This meta-analysis aimed to evaluate the efficacy of catheter ablation in reducing ventricular fibrillation (VF) recurrence in symptomatic BrS compared to standard therapy. **Methods**: Medline, Cochrane Library, and Scopus were systematically searched through 1 June 2025. Study selection, data extraction, and quality assessment were independently conducted by three reviewers. Random-effects meta-analyses were used to pool risk estimates. **Results**: Three studies (two randomized controlled trials, one observational cohort; 130 symptomatic BrS patients) were included. Over a median follow-up of 3.9 years, catheter ablation was associated with a significantly lower risk of VF recurrence compared to standard therapy [risk ratio (RR) = 0.19, 95% confidence interval (CI) = (0.06, 0.60); I^2^ = 36%, p for heterogeneity = 0.21], with no deaths reported in any group. A sensitivity analysis restricted to randomized trials confirmed similar findings in favor of ablation. **Conclusions**: Catheter ablation was associated with reduced VF recurrence compared to ICD therapy alone, supporting its potential role as first-line treatment in symptomatic BrS or as an alternative for patients who decline ICD implantation.

## 1. Introduction

Brugada syndrome (BrS) is a prominent inherited arrhythmogenic disorder and a major cause of sudden cardiac death in individuals without structural heart disease, predominantly due to ventricular fibrillation (VF) [1,2]. The implantable cardioverter-defibrillator (ICD) has traditionally served as the primary therapeutic modality for symptomatic BrS [3], while quinidine remains the sole antiarrhythmic agent with proven efficacy in this context [4]. However, both strategies possess notable limitations. Although ICDs reliably terminate life-threatening arrhythmias, they do not prevent their occurrence and may subject young patients to significant psychological distress due to recurrent shocks. Moreover, long-term ICD use is associated with a spectrum of adverse events, including device-related complications and inappropriate discharges [5,6,7,8]. Management of recurrent VF, particularly in the setting of electrical storm, remains clinically challenging [6,9]. In parallel, the clinical utility of quinidine is hindered by limited global availability and a high incidence of dose-limiting side effects during chronic therapy [10,11].

Seminal investigations have delineated a localized arrhythmogenic substrate situated within the right ventricular (RV) epicardium in patients with BrS, characterized by distinct electrophysiological abnormalities that have emerged as therapeutic targets for catheter ablation [12,13,14,15]. The presence, extent, and functional properties of this substrate have been shown to play a pivotal role in both the symptomatic expression and the arrhythmic trajectory of the disease, thereby constituting a key phenotypic hallmark of BrS [16,17,18,19]. Initial attempts to eliminate these epicardial abnormalities laid the foundation for substrate-guided ablation strategies. Subsequent clinical experience has demonstrated that radiofrequency catheter ablation can markedly reduce VF recurrence, leading to its growing recognition as a viable disease-modifying intervention within the electrophysiology community [12].

To date, the evidence supporting catheter ablation in BrS has been primarily derived from case series and small single-arm observational studies evaluating its impact on VF recurrence [14,20,21,22,23,24]. More recently, two large-scale observational cohorts [12,16] with extended follow-up, along with a meta-analysis of non-comparative studies [25], have reinforced the potential efficacy of substrate-guided ablation in symptomatic BrS. However, studies directly comparing catheter ablation with conventional ICD therapy remain limited. In light of emerging comparative data [26,27,28], a meta-analysis was conducted to evaluate the clinical impact of catheter ablation relative to standard ICD therapy in patients with symptomatic BrS.

## 2. Materials and Methods

This study was designed and executed in accordance with the methodological standards outlined in the Cochrane Handbook for Systematic Reviews of Interventions [29], and its reporting adhered to the Preferred Reporting Items for Systematic Reviews and Meta-Analyses (PRISMA) 2020 framework (Supplementary Table S1) [30]. The protocol was prospectively registered on the Open Science Framework (DOI: 10.17605/OSF.IO/TX4PS) and was implemented as planned, without deviation.

### 2.1. Search Strategy

The search strategy was collaboratively developed by two investigators (P.K. and N.F.), with a comprehensive and independent systematic search subsequently executed by three reviewers. Electronic databases queried included MEDLINE (via PubMed), Scopus, and the Cochrane Database of Systematic Reviews, covering all records from database inception to 1 June 2025. No filters were applied with respect to publication date, language, study status, or year of dissemination. The search syntax integrated both free-text keywords and controlled vocabulary (Medical Subject Headings, MeSHs), focusing on terms such as Brugada syndrome, ablation, and ventricular fibrillation. To maximize retrieval of relevant evidence, supplementary sources were also examined, including manual searches of ClinicalTrials.gov, Epistemonikos, and Google Scholar. Citation mining—both backward and forward—was conducted using the citationchaser package in R to ensure comprehensive coverage of the literature landscape [31]. To ensure transparency and reproducibility of the literature retrieval process, the full search strategies for all databases are presented in Supplementary Tables S2–S4.

### 2.2. Eligibility Criteria

#### 2.2.1. Inclusion Criteria

Eligible studies comprised randomized controlled trials (RCTs) and observational cohorts that evaluated the comparative effectiveness of catheter ablation versus standard ICD therapy in adults (≥18 years) with symptomatic Brugada syndrome.

#### 2.2.2. Exclusion Criteria

Studies were excluded if they met any of the following criteria: (i) single-arm designs, case reports, or case series; (ii) non-original contributions, such as editorials, letters to the editor, commentaries, or expert opinion pieces; (iii) non-peer-reviewed or non-primary research formats including clinical guidelines, conference abstracts, study protocols, or dissertations; and (iv) non-comparative or non-longitudinal designs, such as cross-sectional analyses, case–control studies, or crossover trials.

### 2.3. Outcomes

The primary endpoint of the present meta-analysis was the comparative risk of VF recurrence among individuals with symptomatic BrS undergoing catheter ablation versus standard ICD therapy. The secondary outcomes included all-cause mortality, arrhythmic mortality, appropriate ICD therapies, and major procedural complications. Where applicable, the outcomes were extracted and analyzed according to the definitions and follow-up durations specified in each source study.

### 2.4. Study Selection

During the initial screening phase, three reviewers independently evaluated the titles and abstracts of all records identified through the predefined search strategy. To preserve the inclusiveness of the selection process, no studies were excluded solely because of discordance at this stage. Full-text articles were subsequently assessed in duplicate by the same reviewers, with discrepancies resolved by consensus or, when required, through arbitration by a senior investigator. The screening workflow was supported by the use of Abstrackr [32] for citation review, while reference organization and deduplication were managed using Mendeley.

### 2.5. Data Extraction

A structured data extraction template was developed and iteratively refined through a pilot phase involving a representative subset of four studies. This process included calibration exercises to harmonize reviewer interpretation and optimize consistency. Upon finalization of the standardized form, data extraction was independently performed by three investigators. Any discrepancies were resolved through discussion and consensus, or, when required, in consultation with a senior author.

For each eligible study, data were extracted across two principal domains: (1) study-level characteristics—including design, sample size, ablation technique, primary inclusion criteria, primary endpoint, and duration of follow-up—and (2) baseline patient characteristics—group-specific demographics, such as sample size, proportion of male participants, mean age, prevalence of spontaneous Type 1 ECG pattern, SCN5A mutation positivity, history of sudden cardiac arrest (SCA), and quinidine usage rates. When critical data were unavailable or not explicitly reported, corresponding authors were contacted to request Supplementary Information.

### 2.6. Quality Assessment

The methodological quality of the included studies was critically evaluated by two independent reviewers using validated risk-of-bias assessment tools appropriate to study design. For observational studies, the Risk of Bias in Non-Randomized Studies of Interventions (ROBINS-I) tool [33] was employed, offering a structured framework for assessing bias across domains relevant to non-randomized epidemiologic research. For RCTs, the Cochrane Risk of Bias 2 (RoB 2) tool [34] was utilized to evaluate methodological rigor and potential sources of bias in random sequence generation, allocation concealment, blinding, and outcome assessment. Discrepancies between reviewers were resolved through discussion; when consensus could not be reached, a third reviewer served as an adjudicator.

### 2.7. Data Analysis

All statistical analyses were performed using R Statistical Software (version 4.2). For each included study, risk ratios (RRs) and corresponding 95% confidence intervals (CIs) were calculated based on raw event data, which were consistently available across all comparisons. The catheter ablation group was treated as the exposure of interest, with standard ICD therapy serving as the reference group. Pooled effect estimates were derived using a random-effects model employing a restricted maximum likelihood (REML) estimator to account for between-study variance within a frequentist framework. Statistical significance was defined by a two-sided *p*-value < 0.05. Heterogeneity among studies was evaluated using the I^2^ statistic, interpreted according to conventional thresholds: 0–30% (low), 30–50% (moderate), 50–75% (substantial), and >75% (considerable) [35]. Cochran’s Q test was also applied to assess the presence of statistical heterogeneity. To test the robustness of the findings, a sensitivity analysis was conducted by restricting the meta-analysis to RCTs.

Given the modest number of available comparative studies, subgroup analyses and meta-regression were not performed to preserve analytical robustness. Similarly, assessments of publication bias were not conducted, as these methods are not considered reliable in meta-analyses with a limited study base [36].

## 3. Results

### Study Selection and Characteristics

**Figure 1:**
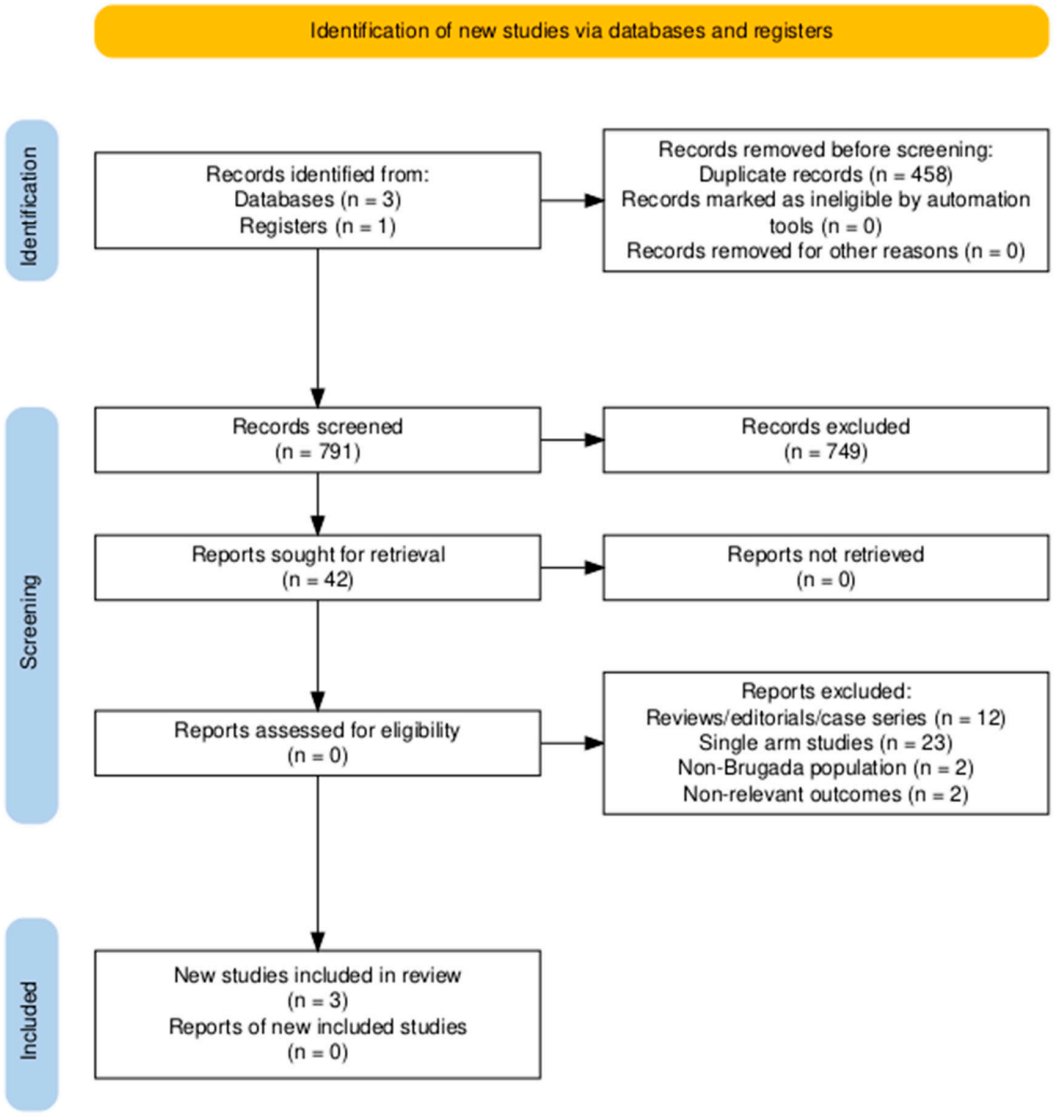
Preferred Reporting Items for Systematic Reviews and Meta-Analyses (PRISMA) flow diagram.

The study selection process is depicted in the PRISMA flow diagram (Figure 1). After duplicate removal, 791 unique records were identified through systematic database searches and screened at the title and abstract level. Of these, 749 were excluded for not meeting the eligibility criteria based on relevance. The remaining 42 full-text articles were assessed in detail, resulting in the inclusion of 3 studies that fulfilled all predefined inclusion criteria [26,27,28].

The principal characteristics of the included studies are summarized in Table 1. Three controlled investigations—comprising two RCTs and one observational cohort—collectively enrolled 130 symptomatic individuals with BrS. The study populations were predominantly male (92%), with mean ages ranging from 38.8 to 47.8 years. The median follow-up duration was 3.9 years (interquartile range: 3–4 years). All the studies employed an epicardial ablation approach, with one incorporating adjunctive endocardial lesions. A spontaneous Type 1 electrocardiographic pattern was reported in 82% of the patients, while SCN5A mutations were present in 30%. A history of sudden cardiac arrest (SCA) was documented in 78% of the ablation recipients and 73% of the controls. VF events were adjudicated through ICD interrogation in the RCTs [27,28]. In the study by Li et al. [26], catheter ablation was offered to patients who declined ICD implantation, with follow-up conducted systematically through outpatient visits or telephone consultations at 3, 6, and 12 months post-discharge and annually thereafter. At each follow-up, arrhythmia surveillance included 12-lead electrocardiography and 24-h Holter monitoring [26].

**Table 1:** Baseline characteristics of included studies.

Study, Year	Study Design	Follow-Up (Years)	Ablation Approach	Sample Size		Mean Age (Years) ^a^		Male Sex (%)		Spontaneous Type 1 ECG (%)		SCN5A Positive (%)		History of SCA (%)		Quinidine Continuation (%)	
				A	C	A	C	A	C	A	C	A	C	A	C	A	C
Li et al. (2023) [26]	RCT	4	Epicardial	26	14	43.7 ± 12.1	43.7 ± 12.1	83	83	57.7	50	34.6	42.9	100	100	0	38
Nademanee et al. (2025) [27]	RCT	3	Epicardial	25	25	43 ± 14	44 ± 13	100	100	96	84	13	18	96	84	0	0
Pappone et al. (2025) [28]	Observational	3.9	Epicardial ± Endocardial	18	22	38.8 ± 8.9	47.8 ± 8.3	88.9	90.9	100	100	44.4	36.4	22.2	45.5	N/R	N/R

Abbreviations: A, ablation group; C, control group; ECG, electrocardiogram; N/R, not reported; RCT, randomized controlled trial; SCA, sudden cardiac arrest; SCN5A, sodium voltage-gated channel alpha subunit 5 gene. a Reported as mean ± standard deviation.

**Figure 2:**
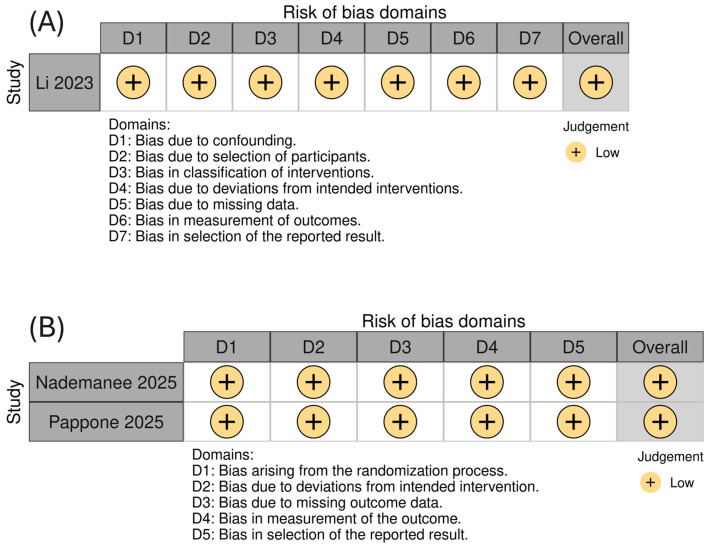
Risk of bias assessment for the included studies using (**A**) the ROBINS-I tool for on-randomized studies and (**B**) the RoB 2.0 tool for randomized controlled trials, presented using a colorblind-friendly scheme.

Methodological appraisal using the RoB 2 tool for randomized trials and the ROBINS-I tool for non-randomized studies indicated a uniformly low risk of bias across all included studies (Figure 2).

#### Ventricular Fibrillation

**Figure 3:**
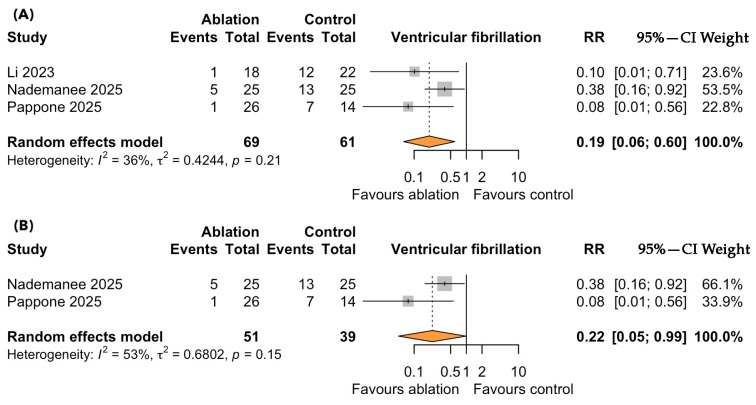
Forest plots depicting the effect of catheter ablation versus standard ICD therapy on ventricular fibrillation (VF) recurrence in patients with symptomatic Brugada syndrome: (**A**) overall analysis including all eligible studies; (**B**) sensitivity analysis restricted to randomized controlled trials. Abbreviations: CI, confidence interval; RR, risk ratio.

A total of three controlled studies, including 130 symptomatic patients with BrS, evaluated the impact of catheter ablation versus standard ICD therapy on the risk of VF. Over a median follow-up of 3.9 years, catheter ablation was associated with a significantly lower risk of VF recurrence compared to standard therapy (RR = 0.19; 95% CI: 0.06–0.60; *p* < 0.001; I^2^ = 36%, p for heterogeneity = 0.21; Figure 3A). The direction and magnitude of the effect were consistent across all the included studies, with no evidence of substantial heterogeneity. A sensitivity analysis restricted to the RCTs yielded similarly significant results, further reinforcing the robustness of the observed association in favor of ablation (Figure 3B).

## 4. Discussion

This is the first meta-analysis of preliminary comparative data assessing the efficacy of catheter ablation versus standard therapy in reducing VF recurrence among symptomatic individuals with BrS. Our findings demonstrate that catheter ablation was associated with a statistically significant 81% relative risk reduction in VF events compared with standard ICD therapy, underscoring its potential as a disease-modifying intervention in this high-risk population.

These results expand upon and complement a recent meta-analysis by Casado Arroyo et al. [25], which pooled outcomes from single-arm observational studies and reported a VF recurrence rate of 3.8% following catheter ablation across a heterogeneous BrS population. Although their findings support the procedural efficacy of ablation, the absence of a comparator arm limited conclusions regarding its incremental benefit over standard therapy. By directly incorporating controlled studies, our meta-analysis provides more robust evidence of the therapeutic advantage conferred by ablation, affirming its utility not merely in arrhythmia suppression but as a strategic alternative to conventional ICD-based management in selected patients.

ICD therapy remains the cornerstone for preventing sudden cardiac death in patients with symptomatic BrS, as endorsed by current international guidelines [37,38]. However, real-world adherence to these recommendations is suboptimal [39]. Across multiple registries and observational studies, ICD utilization among eligible individuals remains markedly limited, with reported rates ranging from 24% in Europe [40] to 30–50% in the United States [41]. In Asia, ICD adoption is even lower, averaging 12% and dropping to as little as 1.5% in countries such as Indonesia [42]. This underutilization could possibly reflect a confluence of factors, including insufficient awareness among clinicians and patients, and procedural concerns. These challenges are further compounded by the recognition that the ICD, while effective in terminating malignant arrhythmias, does not modify the underlying electrophysiologic substrate of BrS. Consequently, there is a growing imperative to investigate alternative therapeutic strategies capable of directly targeting the arrhythmogenic mechanisms in patients who decline or are ineligible for ICD implantation.

A considerable subset of individuals with BrS who undergo ICD implantation confront enduring challenges related to device complications and diminished quality of life [43,44]. While current ESC guidelines endorse ICD therapy as the cornerstone for sudden cardiac death prevention in high-risk BrS populations [3], its acceptance as a universal strategy remains nuanced and, at times, contested [45,46]. Particularly among younger patients, there exists a discernible reluctance to embrace lifelong device dependence—reflecting not only concerns regarding procedural morbidity and long-term complications but also the profound psychosocial toll associated with ICD implantation [43]. Empirical data indicate that ICD recipients frequently report elevated levels of anxiety and depression, coupled with inferior health-related quality-of-life indices when compared to their counterparts managed with catheter ablation [28]. These findings accentuate the imperative to consider a broader, patient-centered framework in BrS management—one that balances arrhythmic risk mitigation with individual values, emotional well-being, and the cumulative burden of chronic device therapy.

Consistent with our findings, a recent 30-year single-center experience by Monaco et al. [47] reinforces the growing role of substrate ablation in the management of high-risk BrS. Among 1206 patients with BrS, 397 (33%) received active treatment—primarily ICD implantation (25.4%), antiarrhythmic drugs (12.4%, including 2.4% with quinidine), or epicardial ablation (5.9%). Over a mean follow-up of approximately 9.5 years, epicardial ablation was associated with a significant 80% reduction in ventricular arrhythmia burden, aligning with the substantial reduction in VF risk observed in our meta-analysis. In contrast, ICD therapy—while central to secondary prevention—was associated with frequent complications, including inappropriate shocks (15.4%) and a high rate of device revisions or lead replacements (27.1%). These findings could support the utility of catheter ablation not only as a potential primary therapeutic modality in highly selected symptomatic BrS patients but also as an adjunctive strategy to reduce arrhythmic burden and device-related morbidity.

### 4.1. Catheter Ablation in BrS Management: Future Perspectives

Building on the accumulating evidence of its efficacy, catheter ablation is increasingly being recognized as a promising therapeutic alternative for symptomatic BrS patients who decline ICD implantation [26,48]. This paradigm is supported by recent data from Li et al. [26], who demonstrated that substrate-targeted ablation may substantially reduce the risk of VF recurrence in high-risk individuals unwilling to undergo device therapy. These findings, consistent with earlier reports and corroborated by our meta-analytic results, highlight the potential of ablation to serve not merely as an adjunct but, in select cases, as a stand-alone strategy for arrhythmic risk mitigation. Nevertheless, such an approach must be approached with caution and should not supplant ICD therapy until validated by large, prospective multicenter trials [12,16].

The implications of these findings extend beyond individual treatment decisions. As seen in other substrate-based arrhythmogenic conditions, such as structural cardiomyopathies [49], earlier intervention may offer superior arrhythmic control and improve quality of life. Analogously, early ablation in BrS may attenuate long-term VF risk and mitigate the psychosocial burden associated with chronic ICD therapy, particularly in younger patients. This hypothesis, however, remains to be substantiated through rigorous clinical investigation aimed at identifying patients most likely to benefit from early substrate modification.

Despite encouraging outcomes, it is critical to acknowledge that the generalizability of catheter ablation remains limited by procedural complexity, operator expertise, and center-specific experience. Ablation, when suboptimally executed, may incur substantial risk, and current outcomes may not be replicable in less specialized environments [50]. At present, catheter ablation should be viewed as a complementary tool to ICD therapy, with the potential to enhance clinical outcomes and patient-reported quality of life. Future research should prioritize the delineation of patient selection criteria, procedural standardization, and the integration of ablation into comprehensive BrS management algorithms that do not compromise safety.

### 4.2. Strengths and Limitations

Despite the methodological rigor of this meta-analysis, several limitations merit acknowledgment. First, the analysis was conducted at the study level without access to individual participant data, limiting the ability to assess effect modification or perform stratified analyses based on patient-specific characteristics. However, the predominance of RCTs likely mitigated baseline imbalances between treatment arms, and the observed low-to-moderate statistical heterogeneity supports the consistency of treatment effects across studies. Second, data on important secondary outcomes—including all-cause mortality, arrhythmic mortality, appropriate ICD interventions, quality of life assessment, and major procedural complications—were inconsistently reported, precluding formal quantitative synthesis. Notably, no deaths were reported in any study throughout the follow-up period. Third, safety data were limited; however, the few reported complications (two cases of pericarditis and two of hemopericardium) reflect an event rate comparable to that observed in general ablation populations [51] and were not associated with adverse clinical sequelae. Fourth, the use of adjunctive pharmacologic therapy, particularly quinidine, was not standardized across studies and was generally left to the discretion of the treating physician. While this reflects real-world practice, it introduces a degree of therapeutic variability that may have influenced the outcomes. Finally, while the findings support the efficacy of catheter ablation in symptomatic BrS, their generalizability to emerging technologies such as pulsed field ablation remains uncertain, as all included studies employed radiofrequency energy delivery.

## 5. Conclusions

In symptomatic BrS, catheter ablation was associated with a significant reduction in VF recurrence compared to standard ICD therapy. These findings support its consideration as a viable alternative in selected high-risk patients, particularly those who decline or are ineligible for ICD implantation. Validation through larger, multicenter trials is warranted to define its role in routine BrS management.

## Data Availability

The data underlying this article will be shared on reasonable request to the corresponding author.

## Supplementary Materials

The following supporting information can be downloaded at: https://www.mdpi.com/article/10.3390/medsci13030115/s1, Table S1: PRISMA 2020 Checklist; Table S2: Search strategy for MEDLINE (Pubmed); Table S3: Search strategy for Cochrane library; Table S4: Search strategy for Scopus.

## References

[1] Wilde A.A.M., Antzelevitch C., Borggrefe M., Brugada J., Brugada R., Brugada P., Corrado D., Hauer R.N., Kass R.S., Nademanee K. (2002). Proposed diagnostic criteria for the Brugada syndrome: Consensus report. Circulation.

[2] Antzelevitch C., Brugada P., Borggrefe M., Brugada J., Brugada R., Corrado D., Gussak I., LeMarec H., Nademanee K., Riera A.R.P. (2005). Brugada syndrome: Report of the second consensus conference: Endorsed by the Heart Rhythm Society and the European Heart Rhythm Association. Circulation.

[3] Zeppenfeld K., Tfelt-Hansen J., de Riva M., Winkel B.G., Behr E.R., A Blom N., Charron P., Corrado D., Dagres N., de Chillou C. (2022). 2022 ESC Guidelines for the management of patients with ventricular arrhythmias and the prevention of sudden cardiac death. Eur. Heart J..

[4] Belhassen B., Glick A., Viskin S. (2004). Efficacy of quinidine in high-risk patients with Brugada syndrome. Circulation.

[5] Olde Nordkamp L.R.A., Postema P.G., Knops R.E., van Dijk N., Limpens J., Wilde A.A.M., de Groot J.R. (2016). Implantable cardioverter-defibrillator harm in young patients with inherited arrhythmia syndromes: A systematic review and meta-analysis of inappropriate shocks and complications. Hear. Rhythm..

[6] Hernandez-Ojeda J., Arbelo E., Borras R., Berne P., Tolosana J.M., Gomez-Juanatey A., Berruezo A., Campuzano O., Sarquella-Brugada G., Mont L. (2017). Patients with Brugada Syndrome and Implanted Cardioverter-Defibrillators: Long-Term Follow-Up. J. Am. Coll. Cardiol..

[7] Sarkozy A., Boussy T., Kourgiannides G., Chierchia G.-B., Richter S., De Potter T., Geelen P., Wellens F., Spreeuwenberg M.D., Brugada P. (2007). Long-term follow-up of primary prophylactic implantable cardioverter-defibrillator therapy in Brugada syndrome. Eur. Heart J..

[8] Sacher F., Probst V., Iesaka Y., Jacon P., Laborderie J., Mizon-Gérard F., Mabo P., Reuter S., Lamaison D., Takahashi Y. (2006). Outcome after implantation of a cardioverter-defibrillator in patients with Brugada syndrome: A multicenter study. Circulation.

[9] Lenarczyk R., Zeppenfeld K., Tfelt-Hansen J., Heinzel F.R., Deneke T., Ene E., Meyer C., Wilde A., Arbelo E., Jędrzejczyk-Patej E. (2024). Management of patients with an electrical storm or clustered ventricular arrhythmias: A clinical consensus statement of the European Heart Rhythm Association of the ESC-endorsed by the Asia-Pacific Heart Rhythm Society, Heart Rhythm Society, and Latin-American Heart Rhythm Society. Europace.

[10] Mazzanti A., Tenuta E., Marino M., Pagan E., Morini M., Memmi M., Colombi B., Tibollo V., Frassoni S., Curcio A. (2019). Low-Dose Quinidine in Brugada Syndrome. Circ. Arrhythmia Electrophysiol..

[11] Viskin S., Wilde A.A.M., Guevara-Valdivia M.E., Daoulah A., Krahn A.D., Zipes D.P., Halkin A., Shivkumar K., Boyle N.G., Adler A. (2013). Quinidine, a life-saving medication for Brugada syndrome, is inaccessible in many countries. J. Am. Coll. Cardiol..

[12] Nademanee K., Chung F.-P., Sacher F., Nogami A., Nakagawa H., Jiang C., Hocini M., Behr E., Veerakul G., Smit J.J. (2023). Long-Term Outcomes of Brugada Substrate Ablation: A Report from BRAVO (Brugada Ablation of VF Substrate Ongoing Multicenter Registry). Circulation.

[13] Pappone C., Brugada J., Vicedomini G., Ciconte G., Manguso F., Saviano M., Vitale R., Cuko A., Giannelli L., Calovic Z. (2017). Electrical Substrate Elimination in 135 Consecutive Patients with Brugada Syndrome. Circ. Arrhythm. Electrophysiol..

[14] Brugada J., Pappone C., Berruezo A., Vicedomini G., Manguso F., Ciconte G., Giannelli L., Santinelli V. (2015). Brugada Syndrome Phenotype Elimination by Epicardial Substrate Ablation. Circ. Arrhythm. Electrophysiol..

[15] Nademanee K., Veerakul G., Chandanamattha P., Chaothawee L., Ariyachaipanich A., Jirasirirojanakorn K., Likittanasombat K., Bhuripanyo K., Ngarmukos T. (2011). Prevention of ventricular fibrillation episodes in Brugada syndrome by catheter ablation over the anterior right ventricular outflow tract epicardium. Circulation.

[16] Santinelli V., Ciconte G., Manguso F., Anastasia L., Micaglio E., Calovic Z., Vicedomini G., Mazza B., Vecchi M., Mecarocci V. (2023). High-risk Brugada syndrome: Factors associated with arrhythmia recurrence and benefits of epicardial ablation in addition to implantable cardioverter defibrillator implantation. Europace.

[17] Pappone C., Ciconte G., Manguso F., Vicedomini G., Mecarocci V., Conti M., Giannelli L., Pozzi P., Borrelli V., Menicanti L. (2018). Assessing the Malignant Ventricular Arrhythmic Substrate in Patients with Brugada Syndrome. J. Am. Coll. Cardiol..

[18] Nademanee K., Haissaguerre M., Hocini M., Nogami A., Cheniti G., Duchateau J., Behr E.R., Saba M., Bokan R., Lou Q. (2019). Mapping and Ablation of Ventricular Fibrillation Associated with Early Repolarization Syndrome. Circulation.

[19] Ciconte G., Monasky M.M., Santinelli V., Micaglio E., Vicedomini G., Anastasia L., Negro G., Borrelli V., Giannelli L., Santini F. (2021). Brugada syndrome genetics is associated with phenotype severity. Eur. Heart J..

[20] Kamakura T., Cochet H., Juhoor M., Nakatani Y., Ramirez F.D., André C., Nakashima T., Krisai P., Takagi T., Tixier R. (2021). Role of endocardial ablation in eliminating an epicardial arrhythmogenic substrate in patients with Brugada syndrome. Heart Rhythm.

[21] Salghetti F., de Asmundis C., Sieira J., Coutiño H.E., Abugattas J.P., Varnavas V., Maj R., Terasawa M., Osório T.G., Stroker E. (2019). Hybrid thoracoscopic epicardial ablation of right ventricular outflow tract in patients with Brugada syndrome. Heart Rhythm.

[22] Mamiya K., Inden Y., Yanagisawa S., Fujii A., Tomomatsu T., Okamoto H., Riku S., Suga K., Furui K., Nakagomi T. (2021). Dynamic Changes in Electrocardiogram Parameters After Epicardial Substrate Catheter Ablation of Brugada Syndrome. Circ. J..

[23] Haanschoten D.M., Elvan A., Postema P.G., Smit J.J.J., Adiyaman A., Ter Bekke R.M.A., Asaad N., Aanhaanen W.T.J., Misier A.R.R., Delnoy P.P.H.M. (2020). Catheter ablation in highly symptomatic Brugada patients: A Dutch case series. Clin. Res. Cardiol..

[24] Tokioka S., Fukamizu S., Kitamura T., Miyazawa S., Kawamura I., Hojo R., Sakurada H., Hiraoka M. (2020). Catheter ablation for monomorphic ventricular tachycardia in Brugada syndrome patients: Detailed characteristics and long-term follow-up. J. Interv. Card. Electrophysiol. Int. J. Arrhythm. Pacing.

[25] Karlinski Vizentin V., Ferreira Felix I., Pivato da Fonseca R., Bozko Collini M., Pinheiro Braga M.A., Serafim Dagostin C., Vidal Armaganijan L., Ackerman M.J., Dantas Brígido A.R., de Carvalho G.D. (2025). Epicardial substrate ablation in patients with symptomatic Brugada syndrome: An updated systematic review and single-arm meta-analysis. Hear. Rhythm..

[26] Li L., Ding L., Zhou L., Wu L., Zheng L., Zhang Z., Xiong Y., Zhang Z., Yao Y. (2023). Outcomes of catheter ablation in high-risk patients with Brugada syndrome refusing an implantable cardioverter defibrillator implantation. Europace.

[27] Nademanee K., Wongcharoen W., Chimparlee N., Chokesuwattanaskul R., Annueypol M., Phusunti K., Sahasatas D., Prechawat S., Prasertwitayakij N., Makarawate P. (2025). Brugada Syndrome Ablation for the Prevention of Ventricular Fibrillation Episodes (BRAVE). Heart Rhythm.

[28] Pappone C., Ciconte G., Vicedomini G., Micaglio E., Boccellino A., Negro G., Giannelli L., Rondine R., Creo P., Tarantino A. (2025). Epicardial ablation in high-risk Brugada syndrome to prevent ventricular fibrillation: Results from a randomized clinical trial. Europace.

[29] Cochrane Handbook for Systematic Reviews of Interventions|Cochrane Training n.d. https://training.cochrane.org/handbook/current.

[30] Page M.J., McKenzie J.E., Bossuyt P.M., Boutron I., Hoffmann T.C., Mulrow C.D., Shamseer L., Tetzlaff J.M., Akl E.A., Brennan S.E. (2021). The PRISMA 2020 statement: An updated guideline for reporting systematic reviews. BMJ.

[31] Haddaway N.R., Grainger M.J., Gray C.T. (2021). Citationchaser: An R Package and Shiny App for Forward and Backward Citations Chasing in Academic Searching. https://zenodo.org/records/4543513.

[32] Wallace B.C., Small K., Brodley C.E., Lau J., Trikalinos T.A. Deploying an interactive machine learning system in an Evidence-based Practice Center: Abstrackr. Proceedings of the IHI’12—ACM SIGHIT International Health Informatics Symposium.

[33] Sterne J.A., Hernán M.A., Reeves B.C., Savović J., Berkman N.D., Viswanathan M., Henry D., Altman D.G., Ansari M.T., Boutron I. (2016). ROBINS-I: A tool for assessing risk of bias in non-randomised studies of interventions. BMJ.

[34] Sterne J.A.C., Savović J., Page M.J., Elbers R.G., Blencowe N.S., Boutron I., Cates C.J., Cheng H.Y., Corbett M.S., Eldridge S.M. (2019). RoB 2: A revised tool for assessing risk of bias in randomised trials. BMJ.

[35] Chapter 10: Analysing Data and Undertaking Meta-Analyses|Cochrane Training n.d. https://training.cochrane.org/handbook/current/chapter-10#section-10-10.

[36] Chapter 13: Assessing Risk of Bias due to Missing Evidence in a Meta-Analysis|Cochrane Training n.d. https://training.cochrane.org/handbook/current/chapter-13#section-13-3-5-6.

[37] Könemann H., Dagres N., Merino J.L., Sticherling C., Zeppenfeld K., Tfelt-Hansen J., Eckardt L. (2023). Spotlight on the 2022 ESC guideline management of ventricular arrhythmias and prevention of sudden cardiac death: 10 novel key aspects. Europace.

[38] Tfelt-Hansen J., Winkel B.G., de Riva M., Zeppenfeld K. (2023). The “10 commandments” for the 2022 ESC Guidelines for the management of patients with ventricular arrhythmias and the prevention of sudden cardiac death. Eur. Heart J..

[39] Conte G., Scherr D., Lenarczyk R., Gandjbachkh E., Boulé S., Spartalis M.D., Behr E.R., Wilde A., Potpara T. (2020). Diagnosis, family screening, and treatment of inherited arrhythmogenic diseases in Europe: Results of the European Heart Rhythm Association Survey. Europace.

[40] John Camm A., Nisam S. (2010). European utilization of the implantable defibrillator: Has 10 years changed the “enigma”?. Europace.

[41] Hoang A., Shen C., Zheng J., Taylor S., Groh W.J., Rosenman M., Buxton A.E., Chen P.-S. (2014). Utilization rates of implantable cardioverter-defibrillators for primary prevention of sudden cardiac death: A 2012 calculation for a midwestern health referral region. Heart Rhythm.

[42] Chia Y.M.F., Teng T.-H.K., Tan E.S.J., Tay W.T., Richards A.M., Chin C.W.L., Shimizu W., Park S.W., Hung C.-L., Ling L.H. (2017). Disparity Between Indications for and Utilization of Implantable Cardioverter Defibrillators in Asian Patients with Heart Failure. Circ. Cardiovasc. Qual. Outcomes.

[43] Probst V., Plassard-Kerdoncuf D., Mansourati J., Mabo P., Sacher F., Fruchet C., Babuty D., Lande G., Guyomarc’H B., Le Marec H. (2011). The psychological impact of implantable cardioverter defibrillator implantation on Brugada syndrome patients. Europace.

[44] van der Werf C., Postema P.G. (2023). The psychological impact of receiving a Brugada syndrome diagnosis. Europace.

[45] Jespersen C.H.B., Krøll J., Bhardwaj P., Winkel B.G., Jacobsen P.K., Jøns C., Haarbo J., Kristensen J., Johansen J.B., Philbert B.T. (2023). Severity of Brugada syndrome disease manifestation and risk of new-onset depression or anxiety: A Danish nationwide study. Europace.

[46] Crotti L., Brugada P., Calkins H., Chevalier P., Conte G., Finocchiaro G., Postema P.G., Probst V., Schwartz P.J., Behr E.R. (2023). From gene-discovery to gene-tailored clinical management: 25 years of research in channelopathies and cardiomyopathies. Europace.

[47] Monaco C., Cespon-Fernandez M., Del Monte A., Pannone L., Della Rocca D., Bala G., Stroker E., Almorad A., Sieira J., Sarkozy A. (2025). Long-term outcome and therapeutic evolution in Brugada syndrome: A 30-year single-center experience. EP Eur..

[48] Conte G., Probst V. (2023). Time to consider catheter ablation as an alternative to implantable cardioverter-defibrillator therapy in high-risk patients with Brugada syndrome?. Europace.

[49] Tung R., Xue Y., Chen M., Jiang C., Shatz D.Y., Besser S.A., Hu H., Chung F.-P., Nakahara S., Kim Y.-H. (2022). First-Line Catheter Ablation of Monomorphic Ventricular Tachycardia in Cardiomyopathy Concurrent with Defibrillator Implantation: The PAUSE-SCD Randomized Trial. Circulation.

[50] Matteucci A., Mariani M.V., Sgarra L., Bonanni M., Frazzetto M., La Fazia V.M., Pierucci N., Lavalle C., Pandozi C., Nardi F. (2024). Epicardial Ablation for Arrhythmogenic Disorders in Patients with Brugada Syndrome. Biomedicines.

[51] Benali K., Khairy P., Hammache N., Petzl A., Da Costa A., Verma A., Andrade J.G., Macle L. (2023). Procedure-Related Complications of Catheter Ablation for Atrial Fibrillation. J. Am. Coll. Cardiol..

